# English News Text Recommendation Method Based on Hypergraph Random Walk Label Expansion

**DOI:** 10.1155/2022/1880114

**Published:** 2022-03-18

**Authors:** Zhehao Wang

**Affiliations:** Department of Culture Education, Henan Institute of Economics and Trade, Zhengzhou, Henan 450000, China

## Abstract

With the rapid development of multimedia and Internet technology, English news text summary technology has received widespread attention as a way to quickly obtain English news text content. The existing method of English news text summarization based on graph model usually takes English news text as the vertices of the graph, and the relationship between the two vertices is represented by the edge. Although it has achieved good results, it cannot quickly sort out the English Complex relationships between news texts. In order to solve this problem, this paper uses the hypergraph model to model the relationship between English news text, and conducts in-depth research on the application of the hypergraph model in the field of English news text summarization. The high popularity of the Internet has brought about earth-shaking changes to the news industry, which makes the news on the Internet a great way for netizens to get news. However, the public cannot pick out satisfactory events from a large amount of news. In order to solve this problem, news event discovery technology that can help users quickly discover and understand hot news is produced. In addition, user personalized recommendation technology will rely on customer operation habits to provide customers with hot events of interest. The personalized news recommendation method adopted in this article has the advantages of integrating discovery and personalized news recommendation, and provides users with a better experience. Compared with the traditional hierarchical clustering algorithm, the algorithm proposed in this paper significantly improves the accuracy.

## 1. Introduction

This paper adopts a new algorithm of mixed recommendation news. This new algorithm uses two methods to conduct experiments to understand the user's interest model [1]. First, understand the multiple characteristics of the event and combine the user's browsing process to form a new model [2]. Secondly, build a model of the news headlines that the user has browsed, and then find groups with similar similarities for users, and then adopt a model that combines SVM and LDA models [3]. Finally, provide news users with news obtained after a series of modeling and calculations. What will appear in the user's hands will be news that is very close to the user's psychology and can quickly satisfy the user's reading experience on time [4]. Based on the needs of the real society, a personalized news event recommendation system is developed in this article [5]. Empirical research shows that the system can achieve the purpose of discovering and recommending news events for users according to preset functions at a stable and efficient operating speed [6]. The first is the proposal of an English news text summarization algorithm based on hypergraph sorting. The HGRVS algorithm includes four parts: extracting features of English news text, constructing hypergraphs, classifying text, and generating summaries. The core of the HGRVS algorithm is to first use the hypergraph sorting algorithm to classify English news text, and then select the items that need to be used from the already classified categories for backup, and compare the selected items above with other news relevance. And remove redundant parts to maintain complete coverage of news events [7]. Secondly, an English news text summarization algorithm based on hypergraph random walk is proposed. This algorithm calculates the stable distribution probability of each English news text through random walks, so as to achieve the purpose of ranking the importance of English news texts [8]. The more important the English news text, the greater the probability of its stable distribution. Then referring to the above conclusions, you need to select the English news text with the highest ranking to perform the de-redundancy operation, and then get the static English news text summary. Finally, after a large number of experiments and a large number of comparisons, the results further verify the effectiveness and advancement of the algorithm [9]. The first method in this article builds a user's interest model based on the user's behavior and habits and integrates the multiple characteristics of the event. The second method is to build a new model based on the news headlines the user has browsed to quickly obtain the user's reading preferences [10].

## 2. Related Works

Literature [11] shows that the Internet and informatization have gradually entered people's lives. Similarly, among the many ways people obtain information, the network has become particularly important. In the world of network information explosion, the problem of information overload is becoming more and more serious, making it impossible for network users to quickly and accurately find information that is of real use to them in the vast information sea. Literature [12] shows that even though scholars have proposed three methods to solve the problem of information overload, search engines, portal websites and classified websites, they cannot solve the user's problem. Literature [13] shows that current users are facing the problem of finding answers to questions and obtaining recommended content of interest. The emergence of keywords provided by search engines provides users with directions for finding answers to questions, but a few simple keywords. But it is difficult to bear the user's complete idea, so the blind search by the user leads to inefficient browsing. Literature [14] shows that as of February 2020, China's Internet penetration rate has reached 79.8%, and the number of Chinese Internet users has increased significantly. The number of Internet users in 2020 alone will reach 39.75 million. Compared with previous years, the growth rate of Internet users has increased significantly. This shows that the number of users who obtain information by browsing the web has increased rapidly, but these users also face challenges and problems. Nowadays, the mixed news information makes it difficult for Internet users to find articles of interest. Sometimes due to the uneven level of media resources behind the operation of the news website, the focus of website reporting is also different, and some focus on sports events. Some reports are more focused on financial and social aspects. Literature [15] shows that users, as information capturers, will not only pay attention to news in a certain industry. The uneven distribution of website resources causes users to waste time on spanning websites and fail to find all they want to see at one time. From another perspective, most users will choose to read this article after looking for similar news recommended at the end of the article, and users will spend a lot of time searching for such articles. For such problems, incident discovery technology is a powerful solution. This technology will help users sort and classify news, and then find hot events during the period. Literature [16] shows that users can use the saved manual search time to read more articles of interest. Because news has strong effectiveness, the method of static product recommendation on social and business websites cannot meet the two requirements of news popularity and novelty. And for some users, sometimes the public hot events recommended by the event discovery technology cannot catch the user's attention, so you need to provide users with all the content they want to learn about according to their personal interests. And related content personalized recommendation technology. Literature [17] shows that the news events browsed by users in different time periods are related, so it is necessary to improve the personalized design, so that it can promptly eliminate news events that users have read in a short time. Provide users with new hot events at any time. The same problem that needs to be considered in the design is that when there are a large number of visits and news events, the personalized recommendation technology can take a major responsibility, so the development of the performance of the personalized event recommendation system is also facing greater challenge.

## 3. Label Expansion Method Based on Hypergraph Random Walk

### 3.1. Basic Concepts of Hypergraph

In the field of multimedia, a graph model can be used to represent similar things, and two similar images can be connected together to be used in the field of image retrieval. The graph model can describe related things well. But there is a many-to-many relationship in real life. For example, the same author can have multiple papers, and the same paper can have multiple authors. At this time, if the graph model is used to establish connections, the paper is the vertex of the graph. When an author holds two articles, the two articles can be connected by one side. Hypergraph is usually represented by the incidence matrix HVE, which is defined as follows:

(1) $$\begin{array}{c} h\left(v_{i},e_{j}\right)=\left\{\begin{array}{cc} 1, & \text{if}\;v_{i}\in e_{j},\\ 0, & \text{if}\;v_{i}\notin e_{j}. \end{array}\right. \end{array}$$

The relationship between 0 and 1 is used to determine whether a vertex belongs to a hyperedge, and the vertex plays an extremely important role in it. Although the 0, 1 model is simple and easy to use, it causes the problem of missing vertices. Therefore, the probability hypergraph model is used to solve this problem, and its incidence matrix *H* is defined as:

(2) $$\begin{array}{c} h\left(v_{i},e_{j}\right)=\left\{\begin{array}{cc} A\left(i,j\right), & \text{if}\;v_{i}\in e_{j},\\ 0, & \text{if}\;v_{i}\notin e_{j}, \end{array}\right. \end{array}$$

Where *A*(*i*,*j*) is defined as

(3) $$\begin{array}{c} A\left(i,j\right)=e^{-\text{dis}\left(v_{i},v_{j}\right)}. \end{array}$$

It can be seen that the emergence of hypergraph probability model improves the probability of local information and hyperedges. In this way, the relationship between the vertices can be better expressed. In addition, the weight of the superedge is defined as

(4) $$\begin{array}{c} w\left(e_{i}\right)=\sum_{v_{j}\notin e_{i}}A\left(i,j\right). \end{array}$$

The overedge weight matrix *W*_*e*_ is defined as:

(5) $$\begin{array}{c} W_{e}=\left[\begin{array}{cccc} w\left(e_{1}\right) & \; & \; & \;\\ \; & w\left(e_{2}\right) & \; & \;\\ \; & \; & \ldots & \;\\ \; & \; & \; & w\left(e_{n}\right) \end{array}\right]. \end{array}$$

The degree of superedge is defined as:

(6) $$\begin{array}{c} \sigma \left(\text{e}\right)=\sum_{v\in e}h\left(v,e\right). \end{array}$$

The hyperedge matrix *D*_*e*_ is defined as:

(7) $$\begin{array}{c} D_{e}=\left[\begin{array}{cccc} \sigma \left(e_{1}\right) & \; & \; & \;\\ \; & \sigma \left(e_{2}\right) & \; & \;\\ \; & \; & \ldots & \;\\ \; & \; & \; & \sigma \left(e_{n}\right) \end{array}\right]. \end{array}$$

The degree of a vertex is defined as:

(8) $$\begin{array}{c} d\left(\text{v}\right)=\sum_{e\in E}w\left(e\right).h\left(v,e\right). \end{array}$$

The vertex degree matrix *D*_*v*_ is defined as:

(9) $$\begin{array}{c} D_{v}=\left[\begin{array}{cccc} d\left(v_{1}\right) & \; & \; & \;\\ \; & d\left(v_{2}\right) & \; & \;\\ \; & \; & \ldots & \;\\ \; & \; & \; & d\left(v_{n}\right) \end{array}\right]. \end{array}$$

### 3.2. The Construction Method of Hypergraph Model

**Figure 1:**
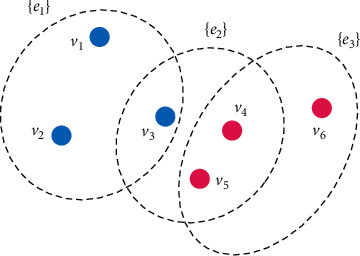
Constructing hypergraph model based on clustering method.

Fuzzy clustering is performed on the vertices of the hypergraph, and then the hyperedges are used to connect various vertices in turn. The number of hyper-edges of the hypergraph is the number of clustering categories, and a hyper-edge center point needs to be determined before using the incidence matrix.  Figure 1 is a schematic diagram of a hypergraph model constructed based on the clustering method.

**Figure 2:**
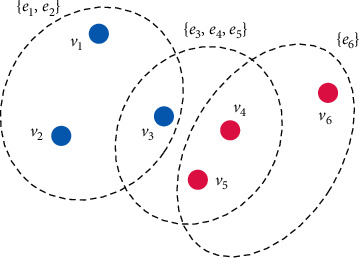
Hypergraph model based on *k*-nearest neighbor method.

Because K-nearest neighbors use hyperedges to connect the vertices near *K* to each vertex, then the number of hyperedges in the hypergraph is the number of vertices. Before calculating the incidence matrix *H*, the center point of the superedge needs to be fixed. For the superedge *e*_1_, the vertex *V*_*i*_ needs to be selected as the center point.  Figure 2 is a hypergraph model diagram constructed based on the *k*-nearest neighbor method.

### 3.3. Hypergraph Sorting Algorithm

The NP problem is transformed into an actual numerical calculation optimization problem, and the loss function is defined as

(10) $$\begin{array}{c} \Omega \left(f\right)=\frac{1}{2}\sum_{e\in E}\sum_{\left(u,v\in e\right)}\frac{w\left(e\right)h\left(u,e\right)h\left(v,e\right)}{\sigma \left(e\right)}{\left(\frac{f\left(u\right)}{\sqrt{d\left(u\right)}}-\frac{f\left(v\right)}{\sqrt{d\left(v\right)}}\right)}^{2}. \end{array}$$

Minimizing the loss function can ensure that the vertices in the same superedge have greater similarity, and the vertices in different superedges are less similar. According to formula (10), the following can be derived:

(11) $$\begin{array}{c} \Omega \left(f\right)=\sum_{e\in E}\sum_{\left(u,v\in e\right)}\frac{w\left(e\right)h\left(u,e\right)h\left(v,e\right)}{\sigma \left(e\right)}\left(\frac{f^{2}\left(u\right)}{\sqrt{d\left(u\right)}}-\frac{f\left(u\right)f\left(v\right)}{\sqrt{d\left(u\right)d\left(v\right)}}\right)\\ =\sum_{u\in V}f^{2}\left(u\right)\sum_{e\in E}\frac{w\left(e\right)h\left(u,e\right)}{d\left(u\right)}\sum_{v\in V}\frac{h\left(v,e\right)}{\sigma \left(e\right)}-\sum_{e\in E}\sum_{\left(u,v\in e\right)}\frac{f\left(u\right)h\left(u,e\right)w\left(e\right)h\left(v,e\right)f\left(v\right)}{\sqrt{d\left(u\right)d\left(v\right)}\sigma \left(e\right)}\\ =f^{T}\left(I-\Theta \right)f. \end{array}$$

If an initialization label vector *y* is given, the closer the score vector *f* to *y* is, the more accurate the calculation is. Therefore, the regular term is defined as:

(12) $$\begin{array}{c} R\left(f\right)={\left\|f-y\right\|}^{2}\\ ={\sum_{u\in V}\left(f\left(u\right)-y\left(u\right)\right)}^{2}. \end{array}$$

The calculated *f* will be solved by the optimal problem:

(13) $$\begin{array}{c} \underset{\int \in R^{\left|v\right|}}{\text{arg min}}\left\{\Omega \left(f\right)+\mu R\left(f\right)\right\}\\ =\underset{\int \in R^{\left|v\right|}}{\text{arg min}}\left\{f^{T}\left(I-\Theta \right)f+\mu {\left\|f-y\right\|}^{2}\right\}. \end{array}$$

Solve by formula (13):

(14) $$\begin{array}{c} f=\left(1-\lambda \right){\left(I-\lambda \Theta \right)}^{-1}y. \end{array}$$

For formula (14), this formula is suitable for the use of the sorting calculation method.

### 3.4. Label Expansion Method

Research scholars sorted the vertices of the hypergraph by the random walk method, which made this method popularized on the hypergraph. The random walk process based on the weighted hypergraph is as follows: First, the starting superpoint is selected according to the superedge weight *w*(*e*) Choose one that contains the current super point *P*. Among the superedges that have been selected, select the transition vertex *v* according to the weight of the super point. Let *P* be the transition probability matrix of a random walk. The calculation method is shown in formula (15):

(15) $$\begin{array}{c} P\left(u,v\right)=\sum_{e\in E}w\left(e\right)\times \frac{h\left(u,e\right)}{\sum_{\hat{e}\in E}w\left(\hat{e}\right)}\times \frac{h_{w}\left(v,e\right)}{\sum_{\hat{v}\in e}h_{w}\left(\hat{v},e\right)}. \end{array}$$

If all nodes are visited, the probability distribution vector will always remain at 1 state. Only under the premise that Markov chains are non-periodic and irreducible can the above objectives be achieved. In order to meet these two conditions, this algorithm completes the random walk of all nodes through the PageRank algorithm under the injection of the mind transfer method. The data transfer in the previous article means that the superpoint located on any superedge may teleport to another superedge with a small probability. It is true that the two super points may not be connected, so it is impossible to transfer them directly. In this paper, this small probability is expressed by a damping factor, as shown in formula (16).

(16) $$\begin{array}{c} v^{i+1}=\left(1-\beta \right)P^{T}v^{i}+\frac{\beta e}{n}. \end{array}$$

## 4. English News Text Recommendation Method Based on Tag Probability Correlation

### 4.1. Probabilistic Correlation Constructs Label Similarity Matrix

Looking at the set of user tags, tags and tags are not completely independent. There is a certain relationship between them. This potential relationship makes each tag different in importance to different users. In addition, the ambiguity of the label will make the label ambiguous in characterizing user characteristics. In part of the label set of user A, the label “apple” contains multiple meanings. It is necessary to clarify whether the label Apple is fruit or an electronic device. The calculation of the correlation between the probability, and then understand the user's tendency to use.

#### 4.1.1. Tag Probability Correlation

From the overall observation, if any two tags are often marked by the user together, then these two tags have a great correlation. From local observations, if a label is marked by the user, the probability of another label being marked by the user is also very high, then it is inferred that the two labels have a strong co-occurrence relationship, as defined in formula (17).

(17) $$\begin{array}{c} p\left(t_{i}|t_{j}\right)=\frac{p\left(t_{i}t_{j}\right)}{p\left(t_{j}\right)}. \end{array}$$

It can be seen from formula (17) that the conditional probability between tags is an asymmetric value. However, there is a symmetrical similar relationship between tags and tags. Therefore, it is necessary to improve the common relationship of tags, and the specific method adopted is shown in formula (18).

(18) $$\begin{array}{c} \text{cor}\left(t_{i},t_{j}\right)=p\left(t_{i}|t_{j}\right)\times p\left(t_{j}|t_{i}\right). \end{array}$$

Combining formulas (17) and (18), the probability correlation between label *f* and label *f* is rewritten as

(19) $$\begin{array}{c} \text{cor}\left(t_{i},t_{j}\right)=\frac{p{\left(t_{i}t_{j}\right)}^{2}}{p\left(t_{i}\right)\times p\left(t_{j}\right)}. \end{array}$$

#### 4.1.2. Label Similarity Matrix

The label matrix is used to characterize users in the vector space model, but traditional algorithms can only reach the level of calculating the similarity between user vectors. Moreover, restricted by the extreme sparseness of user vectors, traditional methods cannot measure the similarity between tags well. Therefore, this paper obtains the probability correlation between tags by calculating the tag correlation phasor. Each tag in the tag set can be represented as a tag correlation vector, defined as formula (20).

(20) $$\begin{array}{c} t_{i}=\left[\text{cor}\left(t_{i},t_{1}\right),\text{cor}\left(t_{i},t_{2}\right),\ldots ,\text{cor}\left(t_{i},t_{n}\right),\ldots ,\text{cor}\left(t_{i},t_{K}\right)\right]. \end{array}$$

Use the formula of cosine similarity to calculate the similarity between tags, as shown in formula (21).

(21) $$\begin{array}{c} \text{sim}\left(t_{1},t_{2}\right)=\frac{t_{1}.t_{2}}{\left\|t_{1}\right\|\times \left\|t_{2}\right\|}=\frac{\sum_{i=1}^{K}\text{cor}\left(t_{1},t_{2}\right)\text{cor}\left(t_{2},t_{1}\right)}{\sqrt{\sum_{i=1}^{K}\text{cor}{\left(t_{1},t_{2}\right)}^{2}}\sqrt{\sum_{i=1}^{K}\text{cor}{\left(t_{2},t_{1}\right)}^{2}}}. \end{array}$$

A similar label matrix S is constructed according to the scheme of label weighting, and then S is used to represent the cosine similarity between the two label phasors. The calculation formula is shown in formula (22).

(22) $$\begin{array}{c} S_{ij}=\left\{\begin{array}{c} 1,\;i=j,\\ \text{sim}\left(t_{i},t_{j}\right),\;i\neq j. \end{array}\right. \end{array}$$

### 4.2. User Tag Weight

The labels that English news users label themselves are distributed as a whole, that is, a small number of representative labels are often labeled, while other personalized labels are usually rarely labeled, which leads to the label weight weighting scheme in the traditional label weight. The probability below is 1. In order to change the problem of repeated results caused by traditional calculation, a label weight weighting scheme is proposed: relevance weight. For English news readers who are tagged after browsing, there is a correlation between the tagged tags on the user. If a certain label of a user has a strong association with any other label, it indicates that the label has a strong identification degree for the user, and the definition is shown in formula (23).

(23) $$\begin{array}{c} \text{cow}\left(u_{j},t_{k}\right)=\frac{\sum_{t_{j}\in u_{i}}\text{cor}\left(t_{k},t_{j}\right)}{\left|u_{i}\right|}. \end{array}$$

Formula (23) only calculates the weight of the label to the user from the local features of the label. A comprehensive label weight must not only take into account the relationship between itself and other labels, but also consider the label to the user from the entire English news collection. The identity of is named inverse user frequency IuF. The specific idea is to use the ratio of the total number of users in the data set to the number of users adding a certain tag and take the logarithm,

(24) $$\begin{array}{c} \text{iuf}\left(t_{k}\right)={\text{log}}_{2}\left(\frac{N}{\text{uf}\left(t_{k}\right)}+1\right). \end{array}$$

Combining the correlation weight and the value of *f*, the weight of the tag in the user *r* is

(25) $$\begin{array}{c} w_{ik}=\text{cow}\left(u_{i,}t_{k}\right)\times \text{iuf}\left(t_{k}\right). \end{array}$$

Considering the probabilistic correlation between tags, a similarity matrix between the old and new tags can be formed, thereby giving users a new experience without creating a sense of alienation from the previous tags. The label matrix can not only remove the limitations of the original matrix, but also contains rich semantic information. Formula (26) is the updated user label matrix.

(26) $$\begin{array}{c} M^{\prime }=M\times S=\left[\begin{array}{cccc} w_{11} & w_{12} & \ldots & w_{1k}\\ w_{21} & w_{22} & \ldots & w_{2k}\\ \ldots & \ldots & \ldots & \ldots \\ w_{N1} & w_{N2} & \ldots & w_{NK} \end{array}\right]\left[\begin{array}{cccc} {\text{sim}}_{11} & {\text{sim}}_{12} & \ldots & {\text{sim}}_{1k}\\ {\text{sim}}_{21} & {\text{sim}}_{22} & \ldots & {\text{sim}}_{2k}\\ \ldots & \ldots & \ldots & \ldots \\ {\text{sim}}_{k1} & {\text{sim}}_{k2} & \ldots & {\text{sim}}_{kk} \end{array}\right]. \end{array}$$

In order to better explain the updated matrix sparse problem, the decomposition matrix *M* is as follows:

(27) $$\begin{array}{c} M^{\prime }=\left[\begin{array}{cccc} w_{11} & w_{12} & \ldots & w_{1k}\\ w_{21} & w_{22} & \ldots & w_{2k}\\ \ldots & \ldots & \ldots & \ldots \\ w_{N1} & w_{N2} & ... & w_{NK} \end{array}\right]\left[\begin{array}{cccc} {\text{sim}}_{11} & {\text{sim}}_{12} & \ldots & {\text{sim}}_{1k}\\ {\text{sim}}_{21} & {\text{sim}}_{22} & \ldots & {\text{sim}}_{2k}\\ \ldots & \ldots & \ldots & \ldots \\ {\text{sim}}_{k1} & {\text{sim}}_{k2} & \ldots & {\text{sim}}_{kk} \end{array}\right]\\ +\left[\begin{array}{cccc} w_{11} & w_{12} & \ldots & w_{1k}\\ w_{21} & w_{22} & \ldots & w_{2k}\\ \ldots & \ldots & \ldots & \ldots \\ w_{N1} & w_{N2} & \ldots & w_{NK} \end{array}\right]\left[\begin{array}{cccc} 0 & {\text{sim}}_{12} & \ldots & {\text{sim}}_{1k}\\ {\text{sim}}_{21} & 0 & \ldots & {\text{sim}}_{2k}\\ \ldots & \ldots & \ldots & \ldots \\ {\text{sim}}_{k1} & {\text{sim}}_{k2} & \ldots & 0 \end{array}\right]. \end{array}$$

Define the ranking function of the recommendation algorithm:

(28) $$\begin{array}{c} f\left(u_{i},d_{p}\right)=\frac{d_{p}.u_{i}}{\left\|d_{p}\right\|\times \left\|u_{i}\right\|}. \end{array}$$

### 4.3. Experimental Operating Environment

System physical operating environment is show in Table 1.

### 4.4. The Influence of Parameter Settings on Method Performance

**Figure 3:**
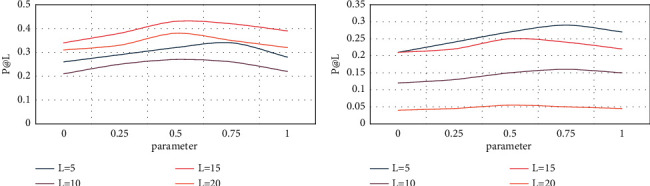
Impact of parameter recommendation algorithm.

The following will examine the influence of parameter ports and thresholds on the algorithm performance in the experiment. When testing the influence of one parameter value on the algorithm, keep the other parameter value unchanged. When weighting the superedge, adjust the ratio of the time factor and the popularity index in English news at any time. The calculation results show that the higher the value, the greater the impact of the time of publishing English news on the extraction of user interest, and the smaller the value, indicating The number of comments and reposts in English news have a low impact on user interest. In this paper, different values of a are set to compare the results of the recommended results at different ports, and the parameter is set to 0.5. When different values are taken, the performance is compared.  Figure 3 shows the experimental results.

**Figure 4:**
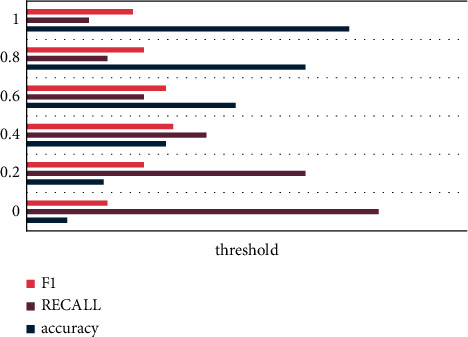
The influence of threshold on recommendation algorithm.

The size of the threshold determines the amount of English news recommended by the recommendation method to users, and different thresholds will have different recommended news numbers. In order to further study the influence of the threshold value on the experimental results, the threshold value is 0.8 for preliminary debugging, and then the threshold value is adjusted according to a certain ratio. The experimental results obtained by the test data set calculation method are shown in Figure 4.

It can be seen from Figure 4 that as the threshold increases, the accuracy of the algorithm gradually increases, while the recall rate of the algorithm gradually decreases.

### 4.5. Comparison of User Label Details and Performance before and after Label Expansion

Due to space limitations, the following is only a comparison of the front and back tags of 10 users as a display, as shown in Table 2.

It can be seen that the reason why these tags are frequently used is that these tags may appear in the user's input items as recommended items, so customers may choose these tags according to their personal ideas, and the second is recommended tags The universal applicability makes people more inclined to directly choose from the existing labels as closely as possible to their own labels. Comparison of different recommendation algorithms as show in Table 3 (Table 3 is reproduced from Huifang et al. [18]).

As can be seen from the table, the LeALpc algorithm proposed in this paper is a higher level of accuracy than the CTR algorithm, UEMR algorithm and BPACMR algorithm from the content perspective, as well as the LPC algorithm, LC and ILCAUSR algorithm from the label perspective. Because these algorithms only consider the needs of users from an overall perspective, but cannot recommend good news based on the user's personal experience, which leads to the problem of low hit rates in the recommendation list. However, the LeALpc algorithm relies on its unique operation mode to provide users with a better experience. It captures the attractiveness of the English news text and user tags to users. Therefore, in real life, the order of recommending news to users is often more important than the news itself. In other evaluation indicators, the LeALpc algorithm is significantly higher than the five algorithms except the ILcAuSR algorithm, but it has no outstanding advantages compared to the ILcAusR algorithm. This is because the IlcAusR algorithm integrates the social relationships between users into the English news recommendation algorithm, which accurately caters to users' interests. But this article has not considered it, which is the direction of this article's future research.

### 4.6. Functional Test and Result Analysis for News Clustering

In order to test the general effect of news clustering, some standards are proposed here, including the accuracy and recall rate of news, *F* value, missed detection rate, false detection rate and normalized detection cost. Event discovery results as show in Table 4.

In this experiment, change the value of *τ* from 0.2 to 0.8 in turn, adjust the step size to 0.1, and observe the changes of *P*_miss_, *P*_*F*_ and (*C*_det_)_norm_ values of each part to determine the optimal *τ* value. The clustering algorithm uses the traditional hierarchy Clustering Algorithm, result as show in Table 5.

**Figure 5:**
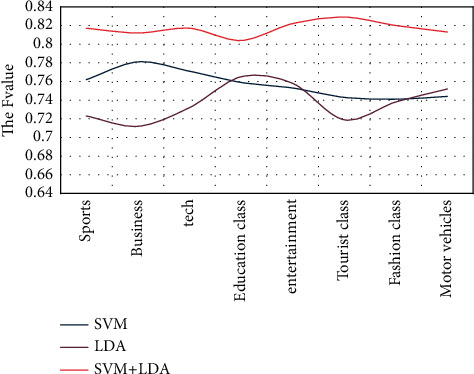
Comparison of each model based on *F* value.

This experiment carried out a comparative analysis experiment on the results of the whole network news data SogouCA obtained from 8 columns in the experimental data corpus of only SVM and LDA and the new model after the hybrid innovation of the two. Finally, it was calculated by the traditional hierarchical clustering algorithm. The final experimental results are shown in Figure 5:

It can be seen from the experimental results that the final *F* value obtained by the two fusion models is more convincing than other models. The SVM and LDA models used in this article are proportionally weighted and the method of calculating model similarity is more convenient And accurate.

The following is an experiment on the proportion of the farthest distance between cluster news and the distance between cluster centers.

According to the data in Table 6, when the proportions of the distance between cluster centers and the farthest distance between clusters are 4/7 and 3/7 respectively, the clustering effect reaches an excellent level. Research on the calculation results found that the larger the proportion of the news distance between clusters, the clusters containing more data points are difficult to merge, so that similar news cannot be merged into the same cluster. If the distance between the cluster centers is large, the difference between the cluster and the cluster. The increase in the number of mergers easily leads to the occurrence of large clusters.

The following is a comparative experiment comparing the execution effect of traditional and improved hierarchical clustering algorithms. In operation, according to the variance between the two algorithms, the accuracy of the experimental calculation data and the closeness of the calculation data sets are estimated, thereby Comparison results in better algorithms. Comparison of two clustering algorithms as show in Table 7.

### 4.7. Functional Test and Result Analysis for News Recommendation

This article uses the relevant data of the CCF big data competition “Analysis of User Browsing News and Personalized News Recommendation” as the experimental data of the recommendation algorithm. These data come from Caijing.com, and the calculated sample is selected as all the news browsing information of 5,000 registered users of the website randomly selected in the second half of 2020. The calculation file has 6 basic attributes, including user number, browsing time, news number, news title, news publication time and its detailed content. 95% of the experimental data is used as the training data set, and 5% as the test data set.

**Figure 6:**
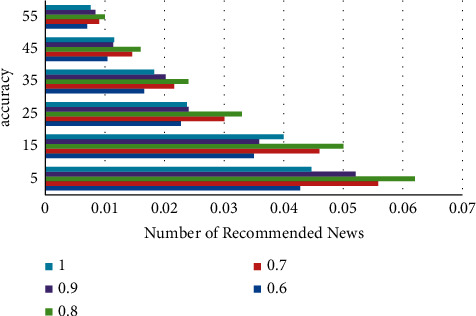
Comparison of accuracy of different parameters *ρ*. According to the experimental results in Figure 6, when the fusion parameter *ρ* = 0.8, the accuracy of the recommendation algorithm is the highest.

In the course of this experiment, the recommendation list is generated using the TopN strategy, that is, the top N news events with the highest ratings are selected and recommended to users. This personalized recommendation does not take the topic model as the key point, but takes the personalized news discovery and recommendation that can capture the user experience on demand as the focus of the evaluation. Therefore, it can be seen that the personalized news recommendation technology used in this article will have a greater advantage in solving the user's cosine similarity. This experiment adjusts the *ρ* value from 0.6 to 1.0 in turn, and compares and analyzes the accuracy of the algorithm recommended in this paper, and obtains the best *ρ* value. The experimental results are shown in Figure 6.

**Figure 7:**
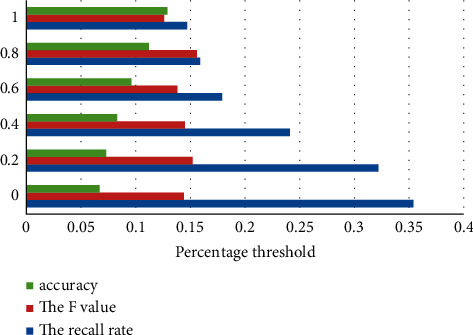
Percentage threshold line chart.

The following is to verify the influence of the percentage threshold on the recommendation result, and calculate the recall rate, accuracy rate and *F* value generated under the use of the recommendation algorithm in this article. The specific representation is shown in Figure 7.

The following experiments are based on the impact of different interest modeling methods on the recommendation results, considering the three basic elements of browsing event time, popularity, and event update frequency to construct a new model. This experiment will use quantitative analysis and chart methods to compare the differences between traditional algorithms and improved algorithms, and then highlight the advantages of the methods described in this article.

**Figure 8:**
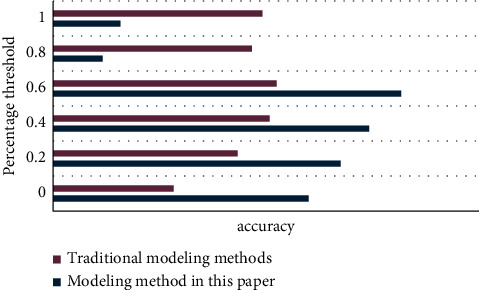
Modeling method accuracy rate line chart.

As can be seen from Figure 8, the modeling method proposed in this article can completely overwhelm the traditional modeling method with huge advantages at all times.

**Figure 9:**
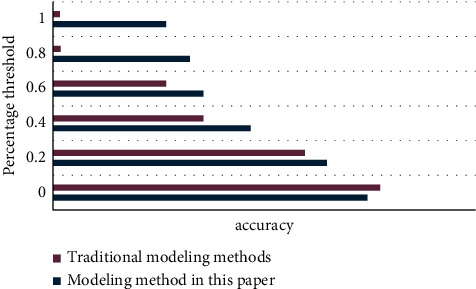
Modeling method recall rate line chart.

As can be seen from Figure 9, the modeling method of this article is significantly better than the traditional modeling method.

**Figure 10:**
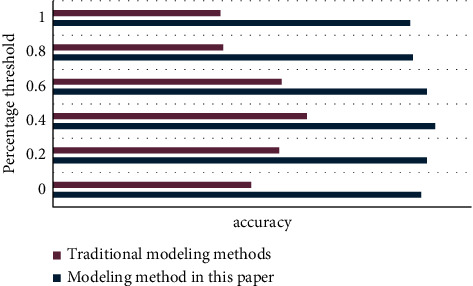
Modeling method *F* value comparison line chart.

As can be seen from the above Figure 10, the modeling method in this article has greatly improved compared with traditional modeling methods in terms of recall rate, accuracy rate, or *F* value. Through the above series of experiments and research, it is completely possible Prove that the improved modeling method has great technical advantages.

**Figure 11:**
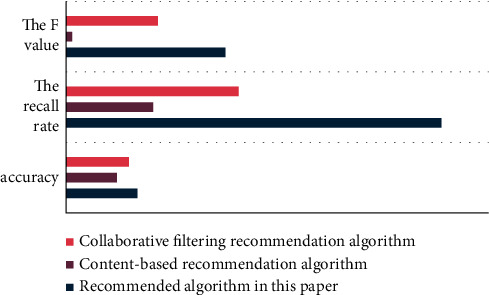
Recommended algorithm comparison histogram.

The following is an experiment that compares the accuracy, recall, and *F* value of the three algorithms in this article, the content-based recommendation algorithm, and the collaborative filtering recommendation algorithm. The comparison results are shown in Figure 11 after calculation:

According to Figure 11, the recommendation algorithm in this paper has more use value than algorithms with other calculation methods in terms of recall rate, accuracy rate or *F* value.

## 5. Conclusion

This paper introduces the hypergraph model into the field of English news text summarization, and proposes an English news text summarization algorithm based on hypergraph sorting and a summary algorithm based on hypergraph random walk. Both algorithms capture the complex relationship between English news texts through the hypergraph model. The HGRVS algorithm uses the idea of hypergraph sorting to classify the English news texts, and the English news texts obtained from the candidates are obtained after a certain calculation The static English text that I want to generate, but RWH uses a hypergraph with random walk characteristics to sort the English text in order, and then form a static English news text summary based on the order. The information is extremely expanded, and it is difficult for Internet users to use only “portal sites and search engines” to find their own suitable news world. However, the technology recommended in this article combines news discovery and personalized recommendation, which can not only help users get news quickly, but also users do not need to worry about and waste time searching for similar news, thus providing users with a better reading experience. The new news recommendation technology proposed in this paper is different from the single recommendation method that only appeared in the previous news recommendation methods. This calculation method first uses the hierarchical clustering algorithm based on the fusion model to collect news events, and then integrates the user's usage and the multiplicity of the events, and calculates the user's interest model, and finally will use the hybrid recommendation algorithm After the calculation, the located news events are sent to the user. After the user reads, the hierarchical clustering of the news interval calculation method is applied to the reading results to calculate more similar articles suitable for the user. This method uses TF-IDF as the basis for similarity calculation, and then expands the calculation of the two topic models, SVM and LDA, with a certain proportion of weighted sum. From the large number of experimental results in this article, comparing the previous unimproved algorithms with the improved algorithm, we can understand that the recommendation algorithm provided in this article has great advantages over other algorithms. To solve the problem of finding similar articles for users, this paper adopts a method for calculating the distance between clusters that can be used for hierarchical clustering. This method adjusts the proportion of the distance between the cluster center and the farthest distance between the clusters in the formula, without affecting the calculation accuracy, and has a great advantage over traditional calculation methods that cannot solve the problem of large clusters.

## Figures and Tables

**Table 1:** System physical operating environment.

Operating environment	Detailed description
Operating system	Win10
Development tools	MyEclipse 10.7
Development language	Java, html, SP, IS, CSS, shell, etc.
Software package	Spring package, lucene package, mongodb package, mysq package
Hardware information	CPU: Intel(R)Core(TM)5–4278 2.6 GHz; RAM: 64.00 GB

**Table 2:** Comparison of user label details before and after label expansion.

English news users	User-added labels before expansion	The label added to the user by the recommendation algorithm after expansion
User-1	Fitness; swimming; health	Health; exercise; exercise; middle-aged
User-2	Beauty; food; design; fashion	Shenzhen; China on the bite of the tongue; movies; reading books
User-3	Painting; collection; history book; poetry	Forbidden city; auction; new media; internet; finance;
User-4	Information; technology; society	Computer; mobile phone; Internet+; innovation; finance; entrepreneurship; e-commerce
User-5	Classical; photography; new media; food; movies; magazines	Art; fashion; struggle; food; media people; entrepreneurship
User-6	Finance; architecture; music	Travel; film; poetry; photography; history; entrepreneurship
User-7	Travel; film; society; struggle	Variety show; fitness; swimming; house; coupons; entrepreneurship
User-8	History; health; health preservation; health care	Collection; health; literature; news; technology; anti-fraud
User-9	Painting; film; art; fashion; freedom; swimming; photography; food	Home; China on the bite of the tongue; new media; entrepreneurship; fitness; technology
User-10	Internet; innovation	E-commerce; finance; entrepreneurship; internet +; technology; struggle; news

**Table 3:** Comparison of different recommendation algorithms.

Name	*L* = 5		*L* = 10		*L* = 15		*L* = 20	
	p	AP	p	AP	p	AP	p	AP
LC	0.315	0.556	0.358	0.425	0.358	0.548	0.286	0.438
LPC	0.306	0.517	0.392	0.486	0.409	0.486	0.245	0.451
ILCAUSR	0.426	0.590	0.455	0.542	0.456	0.592	0.263	0.538
CTR	0.385	0.482	0.412	0.563	0.475	0.575	0.275	0.514
UEMR	0.352	0.476	0.453	0.486	0.446	0.565	0.285	0.495
BPACMR	0.342	0.586	0.393	0.514	0.450	0.541	0.263	0.517
LeALpc	0.338	0.581	0.445	0.599	0.463	0.628	0.272	0.553

**Table 4:** Event discovery results.

Category	Number of related documents	Number of irrelevant documents
The number of documents detected	A	B
Number of documents not detected	C	D

**Table 5:** *P*_miss_, *P*_*F*_, and (*C*_det_)_norm_ changes with *τ*.

	0.2	0.3	0.4	0.5	0.6	0.7	0.8
*P*_miss_	0.0806	0.0763	0.0628	0.0532	0.0452	0.0411	0.0562
*P*_false_	0.0032	0.0026	0.0019	0.0013	0.0011	0.0016	0.0018
(*C*_det_)_norm_	0.8576	0.7112	0.6098	0.6168	0.4563	0.6377	0.7728
*P*_miss_	0.0766	0.0723	0.071	0.0704	0.0655	0.0729	0.0922
*P*_false_	0.0039	0.0033	0.0024	0.002	0.0012	0.0028	0.0038
(*C*_det_)_norm_	0.8983	0.8432	0.7629	0.7423	0.6628	0.8392	0.9927

**Table 6:** Comparison of cluster center distance and proportion.

Weight of distance between cluster centers	The weight of the farthest news distance	Calculation accuracy
0	1	68.87%
1/7	6/7	75.08%
2/7	5/7	81.06%
3/7	4/7	84.68%
4/7	3/7	87.96%
5/7	2/7	72.39%
6/7	1/7	66.16%
1	0	61.67%

**Table 7:** Comparison of two clustering algorithms.

Algorithm name	Accuracy	Within-cluster variance
Traditional hierarchical clustering algorithm	79.28%	0.25956
Improved hierarchical clustering algorithm	88.54%	0.22546

## Data Availability

The data used to support the findings of this study are available from the corresponding author upon request.
